# Synthesis, Characterization, and Evaluation of Redox-Sensitive Chitosan Oligosaccharide Nanoparticles Coated with Phycocyanin for Drug Delivery

**DOI:** 10.1186/s11671-019-3207-4

**Published:** 2019-12-21

**Authors:** Ziting Cheng, Wei Zhang, Xiaoya Hou, Bingjie Wang, Yanping Zhu, Peng Zhang, Feng Zhao, Daquan Chen

**Affiliations:** 1 Collaborative Innovation Center of Advanced Drug Delivery System and Biotech Drugs, Universities of Shandong, Yantai University, Yantai, People’s Republic of China; 2 Department of Radiotherapy, Affiliated Yantai Yuhuangding Hospital of Qingdao University, Yantai, Shandong People’s Republic of China

**Keywords:** Phycocyanin, Biotin, Chitosan oligosaccharide, Nanoparticles, A549 cells, Drug delivery

## Abstract

In this paper, a type of phycocyanin (PC)-functionalized and curcumin (CUR)-loaded biotin-chitosan oligosaccharide-dithiodipropionic acid-curcumin (BCSC) nanoparticles, called CUR-BCSC@PCs, were designed to enhance the biocompatibility of CUR. The structure of BCSC was confirmed using ^1^H-NMR. In CUR-BCSC@PCs with an average hydrodynamic diameter of 160.3 ± 9.0 nm, the biomimetic protein corona gave the nanoparticles excellent stability and the potential to avoid protein adsorption in blood circulation. The in vitro release experiment verified that CUR-BCSC@PCs with redox responsive shells were sensitive to high concentrations of glutathione. In addition, CUR-BCSC@PCs were effective at increasing the inhibitory activity on the proliferation of A549 cells by enhancing the intracellular uptake of CUR. These results indicated that CUR-BCSC@PCs have great application prospects in cancer therapy as effective drug delivery carriers.

## Background

Chitosan oligosaccharide (COS) with the structure of β-(1-4)-linked *D-*glucosamines is a de-polymerization product prepared mainly by deacetylation and enzymatic hydrolysis of chitosan or chitin, which is derived from arthropod exoskeletons or the cell walls of fungi [1, 2]. It is worth noting that heavy metals and dyes can be removed by chitosan and its modified form of materials, in which chitosan acts as an adsorbent [3]. A number of studies have shown that COS possesses several biological properties, such as being anti-cancer, anti-inflammatory, anti-oxidative, and immunostimulatory [4]. COS is considered to be a non-toxic drug delivery carrier material, due to its biocompatibility, high water solubility, and chemical modifiability [5, 6].

In order to improve the solubility of hydrophobic anti-cancer drugs and reduce toxicity to normal tissues, medical researchers have been studying copolymers, which can self-assemble into micelles of a certain particle size range [7, 8]. These co-polymeric assemblies can reach and penetrate the tumor site passively or actively. Micelles are composed of two individual functional sections: the core, in which the hydrophobic drugs are encapsulated, and the outer shell or corona, which controls the in vivo pharmacokinetic properties [8]. Wang et al. [9] grafted deoxycholic acid onto COS chains through a chemical modification to form amphiphilic block copolymers, which could self-assemble into micelles in low-cost inorganic solvents. The hydrophobic core of the micelles contained quercetin, which greatly improved the water solubility of anti-cancer drugs and, in turn, enhanced quercetin’s bioavailability.

Curcumin (CUR) is one of the main chemical constituents of turmeric [10]. In recent years, many researchers have studied the anti-cancer properties of CUR, and many studies have confirmed that this chemical can influence the growth of tumor cells via various signaling pathways and enhance the immune system [11]. In addition, CUR is a photosensitizer with good photodynamic properties [12]. Thus, CUR is a promising drug for cancer therapy. However, its poor solubility, stability, and bioavailability limit its clinical application [13]. To mitigate these disadvantages, many researchers have improved the bioavailability of CUR through drug delivery systems. For example, Chen et al. [14] designed a new type of double pH-sensitive drug carrier, which enhanced the water solubility of CUR and improved its effect on tumor treatment.

Phycocyanin (PC), mainly obtained from cyanobacteria, is known as a water-soluble and light-harvesting pigment protein, which plays a role in capturing and transferring light into chemical energy during photosynthesis [15]. PC exerts multiple biological properties, such as being anti-cancerous [16], anti-inflammatory, and anti-oxidative that have attracted much attention in the fields of food and medicine [15, 17]. Hao et al. [16] discovered that PC inhibited non-small cell lung cancer cell growth by downregulating toll/interleukin-1 receptor domain-containing adaptor protein (TIRAP). In addition, PC is also used in photodynamic therapy (PDT) of tumors as it is an excellent agent with no side effects [18–20]. The utilization of PC as fluorescent probes needs to be conjugated with specific identification elements such as biotin, antibodies, and streptavidin [21, 22]. Biotin, a water-soluble vitamin, is an essential micronutrient in the human body, which has tumor-targeting properties [23]. The biotin-specific receptor proteins, such as avidin, neutravidin, and streptavidin, are highly over-expressed on the surface of several cancer cells compared with that of normal cells [24, 25]. Thus, biotinylated nanoparticles have been extensively studied to be a drug delivery via receptor-mediated endocytosis [24].

**Fig. 1:**
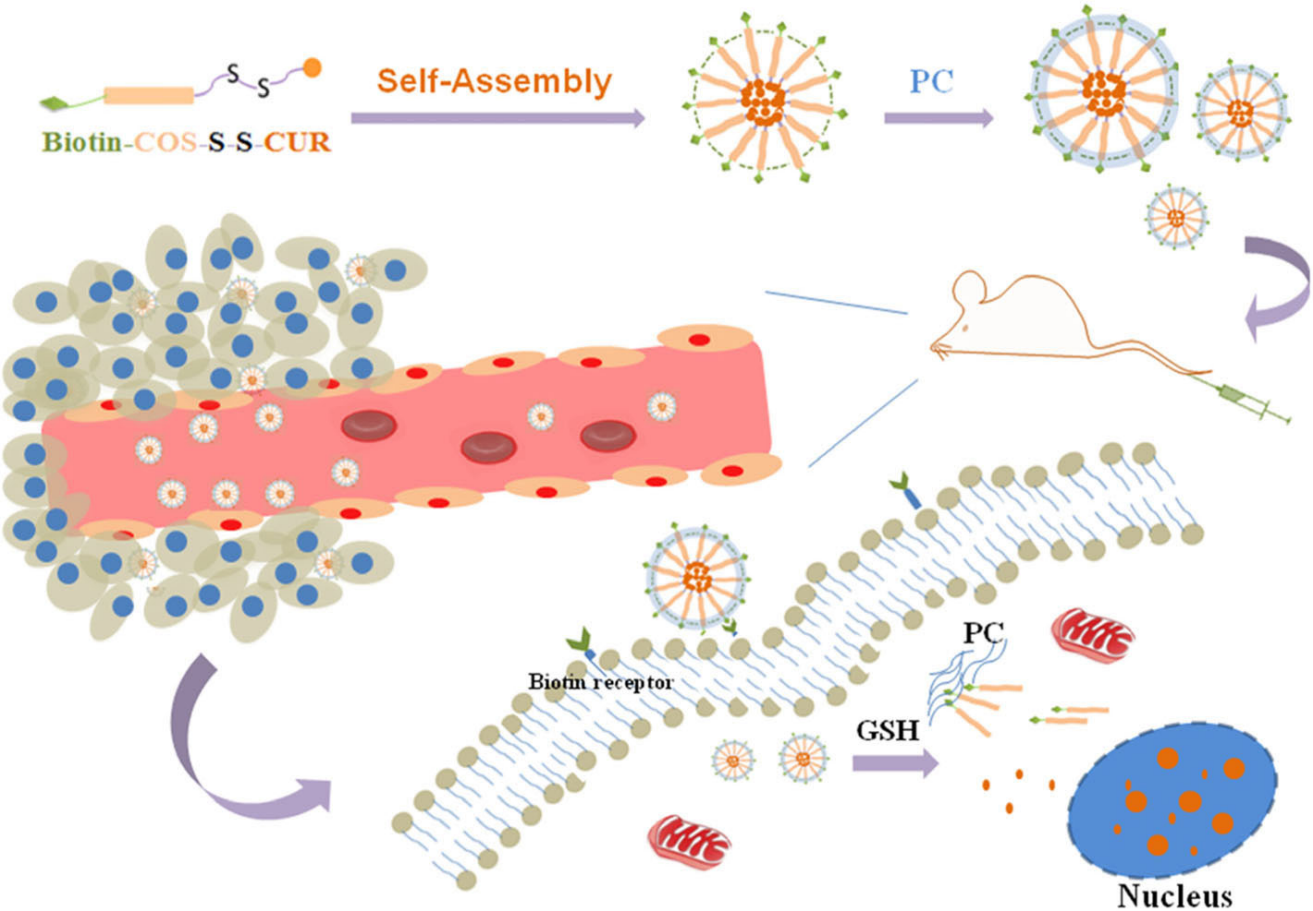
Design and schematic illustration of CUR-BCSC@PCs

In the present study, a novel type of PC functionalized nanocarrier was constructed, and the preparation of CUR-BCSC@PCs is presented in Fig. 1. The disulfide bond between COS and CUR contributed to the reductive sensitivity of the amphiphilic carrier material [26]. CUR was located in the hydrophobic inner core protected by the COS/PC shell. The interaction between biotin and PC and the electrostatic interaction, which was between positively charged COS and negatively charged PC, were used to modify PC onto the surface of CUR-BCSCs. CUR-BCSC@PCs were expected to reach tumor tissues due to improved permeability, the enhanced permeability and retention (EPR) effect, and the biotin receptor targeting effect [27]. In the tumor microenvironment, which has a higher glutathione concentration than normal cells, CUR-BCSC@PCs disintegrated through thiol−disulfide exchange reactions, achieving a high drug concentration in the tumor cells [23, 28, 29]. The CUR-BCSC@PC nano-delivery system described in this paper not only improved the bioavailability of CUR but also has the potential to be effective in the clinical treatment of tumors.

## Materials and Methods

### Materials

CUR was purchased from Zhanyun Chemical Co., Ltd. (Shanghai, China). COS was procured from Shandong Weikang Biomedical Science and Technology Co., Ltd. PC was obtained from Zhejiang Binmei Biotechnology Co., Ltd. 3.3-Dithiodipropionic acid was obtained from Adamas Reagent Co., Ltd. (Shanghai, China). Carbonized carbodiimide hydrochloride (EDCI), dimethylamino pyridine (DMAP), tetrahydrofuran (THF), oxalyl chloride, and biotin were procured from Aladdin Chemistry Co., Ltd. L-Glutathione (GSH) and Hoechst 33342 were acquired from Sigma-Aldrich (Shanghai, China). Dialysis bags (MWCO 300 Da) were obtained from Beijing Biotopped Technology Co., Ltd. Formamide was prepared by Tianjin Fuyu Fine Chemical Co., Ltd. Deionized water was self-made in the laboratory.

An A549 cell line (human lung carcinoma cells) (Bena Culture Collection (Beijing, China)) was chosen to evaluate the cytotoxicity of the novel nanocarrier. A549 cells were grown in DMEM (Saiersi Biotechnology Co., Ltd. (Shangdong, Yantai, China)). Fetal bovine serum (FBS) was obtained from Hyclone (Logan, UT, USA). Penicillin and streptomycin were purchased from Sigma-Aldrich (Shanghai, China). 3-(4,5-dimethyl-2-thiazolyl)-2,5-diphenyl-2-H-tetrazolium bromide (MTT) was also procured from Sigma-Aldrich (Shanghai, China).

### Synthesis of CUR-BCSC@PCs

**Fig. 2:**
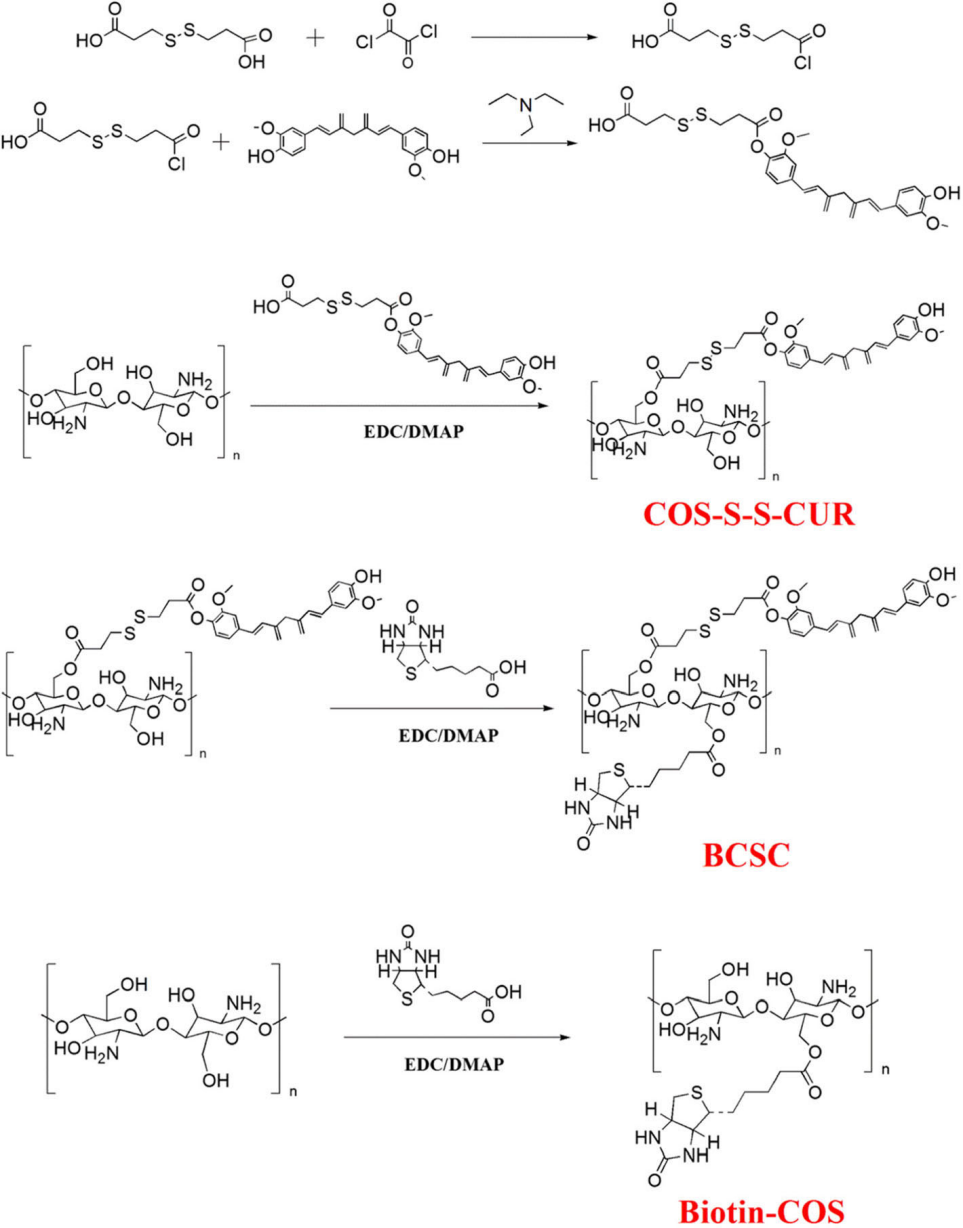
Synthetic route of COS-S-S-CUR, BCSC, and Biotin-COS

The synthetic routes used to prepare CUR-BCSC@PCs are shown in Fig. 2. The detailed experimental procedures are described below.

### Synthesis of COS-S-S-CUR

3.3-Dithiodipropionic acid-functionalized COS was synthesized by esterification catalyzed by acid chloride via a two-step reaction.

Step 1: 3.3-Dithiodipropionic acid (42.05 mg, 0.25 mmol) and dry THF (4 mL) were charged into a brown round-bottom flask, with a stirrer to dissolve the acid. Then, oxalyl chloride (0.3 mmol) diluted with THF was added to a flask, placed in an ice bath. The reaction was maintained at 35 °C. After stirring for 3 h, the unreacted oxalyl chloride was removed by rotary evaporation. Product 1 was prepared by the above steps. CUR was dissolved in 3 mL of THF, containing 34 μL of triethylamine, that was added dropwise to the flask containing product 1 under ice bath conditions and then stirred for 15 min. Subsequently, the mixture was stirred at 50 °C under a nitrogen atmosphere for 6 h. The obtained product was subjected to rotary distillation to remove triethylamine and THF and was then purified by column chromatography to acquire the pure product HOOC-S-S-CUR.

Step 2: The pure product HOOC-S-S-CUR was activated with EDCI (1.2 eq) and DMAP (1.2 eq) in formamide for 2 h. Subsequently, COS dissolved in 4 mL of formamide was added and stirred at 55 °C for 12 h. After the reaction was complete, the solution was dialyzed with a dialysis bag (MWCO 300 Da) and freeze-dried for 12 h.

### Synthesis of BCSC and Biotin-COS

In brief, biotin, EDCI, and DMAP were dissolved in 3 mL of formamide and transferred into a brown round-bottom flask. After stirring for 2 h at 30 °C, COS-S-S-CUR was dissolved in 3 mL of formamide and added dropwise into the flask. The reaction was maintained at 45 °C for 2 days. The final products were dialyzed (MWCO 300 Da) in deionized water and underwent centrifugation and lyophilization to obtain redox-sensitive BCSC. In addition, the synthesis of biotin-COS was conducted by the same method to link biotin to the COS chains.

^1^H-NMR of COS-S-S-CUR, BCSC, and biotin-COS were measured using a mixture of DMSO-D_6_ and D_2_O as the solvent.

### Preparation of CUR-Loaded BCSC Micelles (CUR-BCSCs)

CUR-BCSCs were prepared through the self-assembly method. Ten milligrams of BCSC was dissolved in 4 mL of formamide and then mixed with 1 mL of CUR solution (1 mg/mL) that was dissolved in formamide. The mixed solution was dialyzed using a dialysis bag (MWCO 300 Da) in deionized water for 24 h and the deionized water was changed every 2 h. CUR-BCSCs were filtrated using millipore membranes of 800 nm, 450 nm, and 220 nm.

### Preparation of CUR-BCSC@PCs

The prepared CUR-BCSCs were mixed with an aqueous solution of PC (1.0 mg/mL) and incubated for 30 min at 4 °C. Subsequently, PC was removed using a 100 kDa centrifugal filter and rinsed with water three times. The final product (CUR-BCSC@PC) was stored at 4 °C in darkness for further study.

### Characterizations

Dynamic laser scattering (DLS) measurements were carried out on a Particle Analyzer Delsa Nano C (Beckman Coulter Inc.) to observe the particle size, zeta potential, and polydispersity index (PI). The morphology of CUR-BCSCs and CUR-BCSC@PCs was confirmed by transmission electron microscopy (TEM, H-600; Hitachi, Tokyo, Japan) measurements.

### Encapsulation Efficiency (EE) and Drug Loading Capacity (DL)

HPLC (Agilent 1260GB12C) was used to determine the EE and DL of nanoparticles. First, 2 mL of CUR-BCSCs or CUR-BCSC@PCs was mixed with 3 mL of acetonitrile and demulsified by ultrasound, in which acetonitrile was then added to 10 mL. Before the measurement, the column temperature of the Phenomenex C18 column (250 mm × 4*.*6 mm, 5 um) was adjusted to 25 °C, while the flow rate of the mobile phase was set to 1.0 mL·min^− 1^. The ratio of 0.5% glacial acetic acid to acetonitrile was 40:60 (v/v). In the detection process, with a detection wavelength of 425 nm, 20 μL of samples was injected [30]. The following formula was used to calculate the EE and DL:

$$ \mathrm{EE}\%=\left(\mathrm{Weight}\ \mathrm{of}\ \mathrm{Cur}\ \mathrm{in}\ \mathrm{the}\ \mathrm{nanoparticles}/\mathrm{Weight}\ \mathrm{of}\ \mathrm{the}\ \mathrm{feeding}\ \mathrm{Cur}\right)\times 100\% $$

$$ \mathrm{DL}\%=\left(\mathrm{Mass}\ \mathrm{of}\ \mathrm{Cur}\ \mathrm{in}\ \mathrm{the}\ \mathrm{nanoparticles}/\mathrm{Mass}\ \mathrm{of}\ \mathrm{the}\ \mathrm{nanoparticles}\right)\times 100\% $$

### In vitro Stability Test

Phosphate-buffered saline (PBS) containing GSH (0, 20 μM, 10 mM) was prepared and treated with CUR-BCSC@PCs for 4 h to observe the changes in particle size under different concentrations of glutathione at 37 °C. In addition, the hydrodynamic diameter of CUR-BCSC@PCs was investigated in PBS solution using a Particle Analyzer Delsa Nano C (Beckman Coulter Inc.) at 37 °C at different time points (4, 8, 12, and 24 h).

### In vitro CUR Release from CUR-BCSC@PCs

The in vitro CUR release behaviors of CUR-BCSC@PCs were investigated using the dialysis method. PBS solutions containing glutathione (GSH: 20 μmol/L, 1 mmol/L, 5 mmol/L, 10 mmol/L) were prepared, and 0.5% Tween 80 was added. PBS buffer (45 mL), containing different concentrations of GSH, was added to 50 mL centrifuge tubes; then, a dialysis bag containing 1 mL of CUR-BCSC@PCs was placed in each centrifuge tube, which was shaken at 37 °C. At different time points (0.2, 1, 4, 8, 12, 24, 48, 72 h), 2 mL of release medium was collected, and fresh release medium of the same type was added to keep its volume unchanged. HPLC was used to determine the concentration of CUR in the collected release medium.

### Cell Culture

The human lung carcinoma A549 cells were cultured in DMEM, which included 10% FBS and 1% penicillin-streptomycin, and were incubated at 37 °C in 5% CO_2_ atmosphere [31, 32].

### In vitro Cellular Proliferation Inhibition

The in vitro cytotoxicity of CUR, BCSC micelles (BCSCs), CUR-BCSCs, and CUR-BCSC@PCs against the A549 cell line was evaluated using a standard MTT assay [33]. The A549 cells were seeded in 96-well plates (5000 cells per well) for 24 h to adhere to the wall. The original medium was discarded, and then 100 μL of fresh medium containing free CUR, BCSCs, CUR-BCSCs, and CUR-BCSC@PCs (0.1, 1, 5, 10, 15, and 20 μg/mL based on CUR) was added and cultured for 24 h. Wells without administration were used as blank controls. The cells were subjected to MTT assay by removing the medium and adding 20 μL of MTT solution (5 mg/mL). After incubation for another 4 h at 37 °C in 5% CO_2_ atmosphere, the MTT solution was replaced with 150 μL of DMSO to dissolve the purple MTT-formazan. Subsequently, a microplate reader (Thermo Fisher Scientific Co., Waltham, MA) was used to measure the absorbance of each well at 570 nm.

### In vitro Cell Uptake and Localization

The cellular uptake ability of CUR-BCSCs and CUR-BCSC@PCs was investigated under a fluorescence microscope (FM, Eclipse E400; Nikon Corporation, Tokyo, Japan). A549 cells were seeded in 24-well plates at 4 × 10^4^ cells per well and were co-incubated with CUR-BCSCs and CUR-BCSC@PCs (CUR concentration: 20 μg/mL) at 37 °C in a humidified atmosphere containing 5% CO_2_ for 1, 2, and 4 h. The A549 cells were washed with PBS three times after removing the culture media containing drugs. Then, PBS containing 4% paraformaldehyde was added for 20 min and washed with PBS for another three times. The cell nuclei were stained by Hoechst 33342 for 15 min and observed using an inverted fluorescence microscope.

### Statistical Analysis

All experiments were carried out at least three times and expressed as means ± SD. Statistical tests were analyzed using the Student’s *t* test. *P* < 0.05 was set as statistically significant, and *P* < 0.01 was considered highly significant.

## Results and Discussion

### Characterization of COS, COS-S-S-CUR, Biotin-COS, and BCSC

**Fig. 3:**
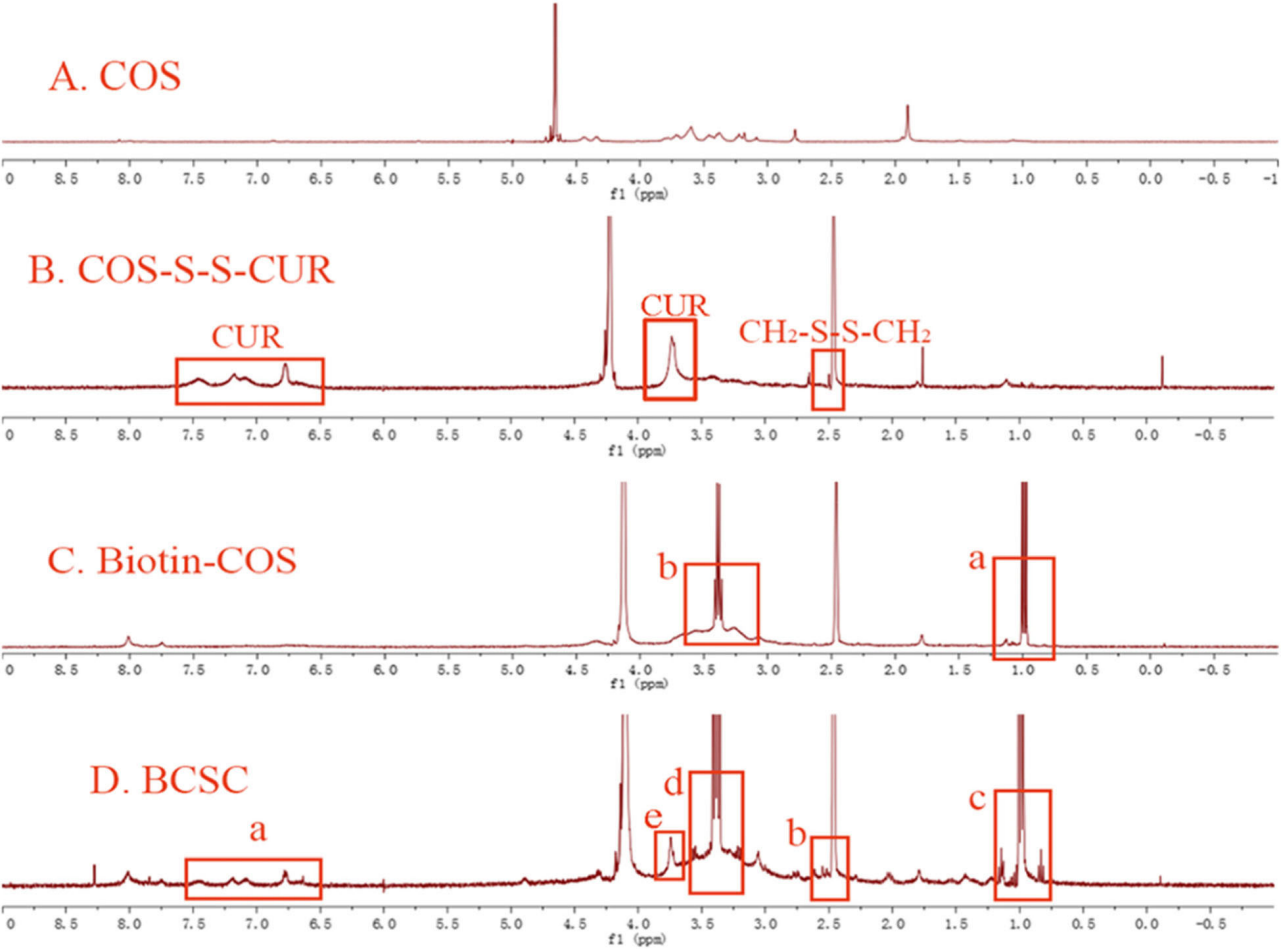
The ^1^H-NMR spectra of COS (**A**), COS-S-S-CUR (**B**), Biotin-COS (**C**), and BCSC (**D**)

The main characteristic resonances of CUR and CH_2_-S-S-CH_2_ appeared on the ^1^H-NMR spectrum of COS-S-S-CUR, proving the successful conjugation of CUR to the COS chains. Compared with the peaks of COS in Fig. 3(A), the characteristic signals of CUR presented in Fig. 3(B) were observed in the region between 6.7 and 7.5 ppm and at 3.75 ppm (−OCH_3_), while the resonance of CH_2_-S-S-CH_2_ at 2.5 ppm was unchanged. As shown in (Fig. 3(C), a, b), the peaks of biotin on the ^1^H-NMR spectrum of Biotin-COS were 0.99 ppm (−CH_2_–) and 3.39 ppm (−CH–S–). ^1^H-NMR spectrum of BCSC is shown in Fig. 3(D). The resonances of CUR (Fig. 3(D), a, e) were seen in the corresponding positions, and the characteristic peak of CH_2_-S-S-CH_2_ (Fig. 3(D), b) was again observed at 2.5 ppm. In addition, the appearance of signals at peak 0.09 ppm and 3.39 ppm (Fig. 3(D), c, d) verified the existence of biotin linked to the COS-S-S-CUR chains. The characteristic resonances of CUR, CH_2_-S-S-CH_2_, and biotin as shown in Fig. 3(D) are consistent with those in Fig. 3(B) and Fig. 3(C), indicating that the amphiphilic material BCSC was synthesized successfully.

### Characterization of CUR-BCSCs and CUR-BCSC@PCs

**Fig. 4:**
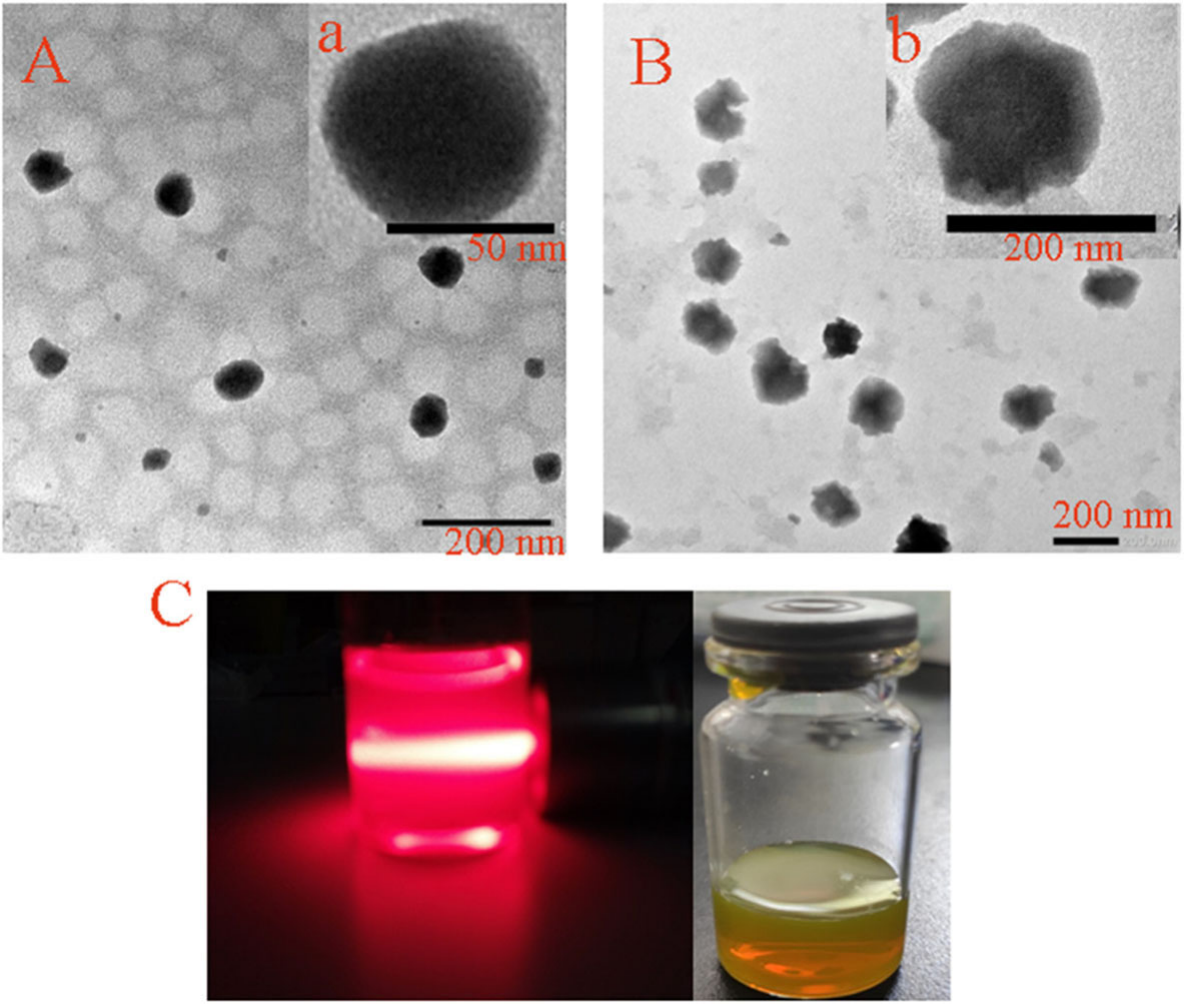
**A** The TEM images of CUR-BCSCs and a single CUR-BCSC. **B** The TEM images of CUR-BCSC@PCs and a single CUR-BCSC@PC. **C** Tyndall effect and the photograph of CUR-BCSC@PCs in water

**Fig. 5:**
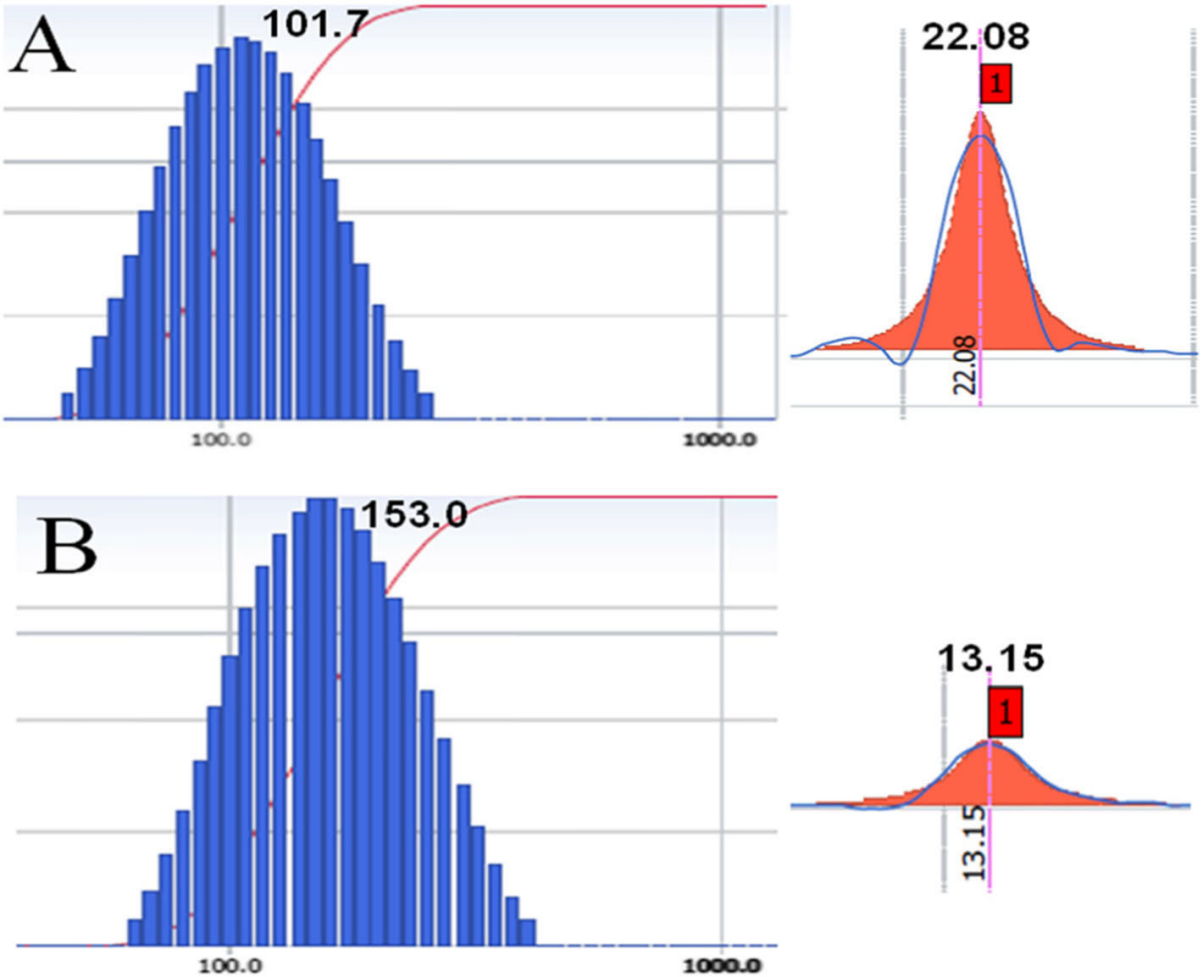
**a** The size distribution and the zeta potential of CUR-BCSCs; **b** The size distribution and the zeta potential of CUR-BCSC@PCs

The morphologies of CUR-BCSCs and CUR-BCSC@PCs were studied by transmission electron microscopy (TEM) (Fig. 4(A, B)). CUR-BCSCs showed a smooth spherical shape (Fig. 4(A), a) under electron microscopy, while CUR-BCSC@PCs possessed an approximately spherical shape with the blooming layer surrounding the CUR-BCSC@PCs (Fig. 4(B), b). This indicated that PC formed a protein corona structure by covering the surfaces of CUR-BCSCs. A clear Tyndall effect of CUR-BCSC@PCs was observed because of the existence of generous nanoparticles (Fig. 4(C)). The particle size, PI, zeta potential, DL (%), and EE (%) of CUR-BCSCs and CUR-BCSC@PCs are illustrated in Table 1. In Fig. 5, the average size of CUR-BCSCs and CUR-BCSC@PCs was 97.8 ± 4.2 nm and 160.3 ± 9.0 nm, respectively. Meanwhile, the PI values of CUR-BCSCs and CUR-BCSC@PCs were 0.181 ± 0.014 and 0.114 ± 0.024, respectively, which are smaller than 0.2, indicating the uniformity of their sizes. The zeta potentials of CUR-BCSCs and CUR-BCSC@PCs were 21.57 ± 0.53 and 12.90 ± 1.93 mV, respectively. Due to the electronegative PC coating, the zeta potential of CUR-BCSCs was higher than that of CUR-BCSC@PCs. The EE of CUR-BCSC@PCs was higher than that of CUR-BCSCs.

**Table 1:** The physiochemical properties of CUR-BCSCs and CUR-BCSC@PCs (*n* = 3)

Preparation	Size (nm)	PI	Zeta (mV)	DL (%)	EE (%)
CUR-BCSCs	97.8 ± 4.2	0.181 ± 0.014	21.57 ± 0.53	7.8 ± 1.08	48.84 ± 7.41
CUR-BCSC@PCs	160.3 ± 9.0	0.114 ± 0.024	12.90 ± 1.93	5.3 ± 0.67	52.24 ± 5.50

### Stability of CUR-BCSC@PCs

**Fig. 6:**
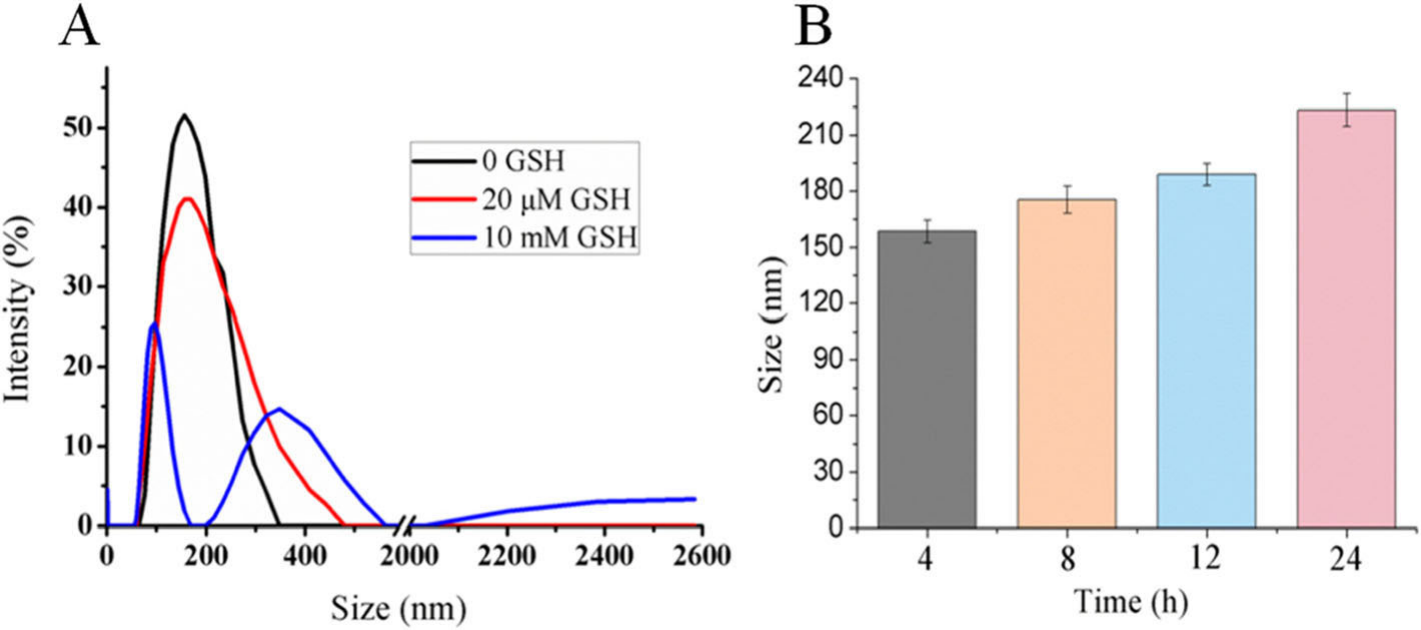
The stability of CUR-BCSC@PCs. **a** Size changes of CUR-BCSC@PCs at different GSH concentration. **b** Size changes of CUR-BCSC@PCs in PBS at different times

As shown in Fig. 6a, due to the reductive nature of the disulfide bond, the bond was cleaved in PBS containing 10 mM GSH, and CUR-BCSC@PCs were disintegrated into polymer fragments, which agglomerated to increase the particle size of the nanoparticles. However, in PBS containing 20 μM GSH, the changes in particle size were minor, which showed a similar result to PBS without GSH. As illustrated in Fig. 6b, particle size was measured at different times to study the stability of CUR-BCSC@PCs in PBS, and the results showed that the particle size of CUR-BCSC@PCs increased slowly over time [14].

### Reduction Response of CUR-BCSC@PCs

**Fig. 7:**
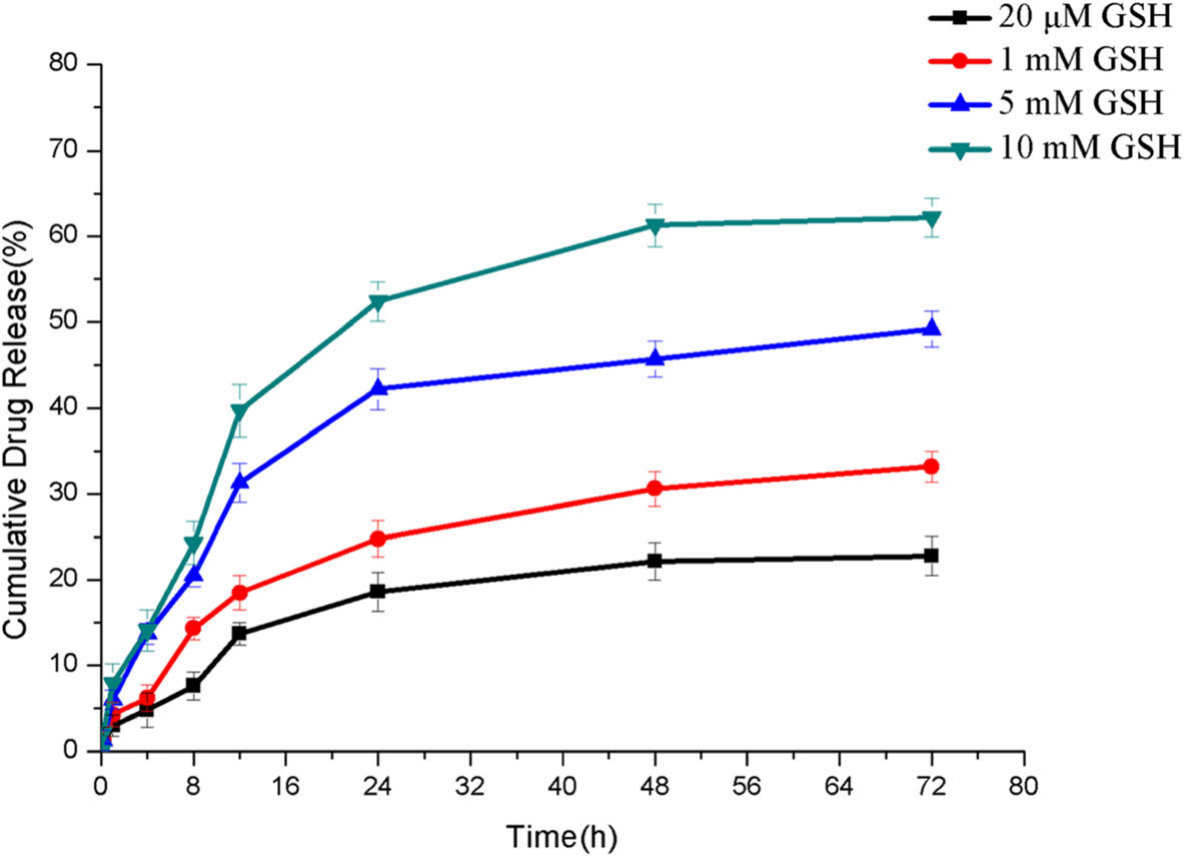
Cumulative release of CUR from CUR-BCSC@PCs at different GSH conditions

It is well established that disulfide bonds are unstable in a tumor-reductive environment. Researchers have used disulfide bonds to connect hydrophilic polymers and hydrophobic drugs to prepare amphiphilic fragments, which can self-assemble in water to form nano-micelles. Then, according to differences in the physiological environment and the tumor environment, disulfide bonds break up at the tumor site to release drugs [34]. In the present study, in vitro drug release was conducted to verify whether CUR-BCSC@PCs could present an expected release property. The reduction response ability of CUR-BCSC@PCs containing disulfide linkages was investigated following the activation of GSH. As shown in Fig. 7, the release of CUR from CUR-BCSC@PCs in the medium with 20 μM GSH at pH 7.4, which simulated the extracellular environment, was extremely slow compared with an environment with 10 mM GSH at pH 7.4. Furthermore, compared with the 20 μM GSH medium, the 1, 5, and 10 mM GSH showed significant differences in release behavior. With the increase in GSH concentration, the release of CUR from CUR-BCSC@PCs was also promoted, indicating that the drug release was in response to the GSH concentration.

### In vitro Cytotoxicity

**Fig. 8:**
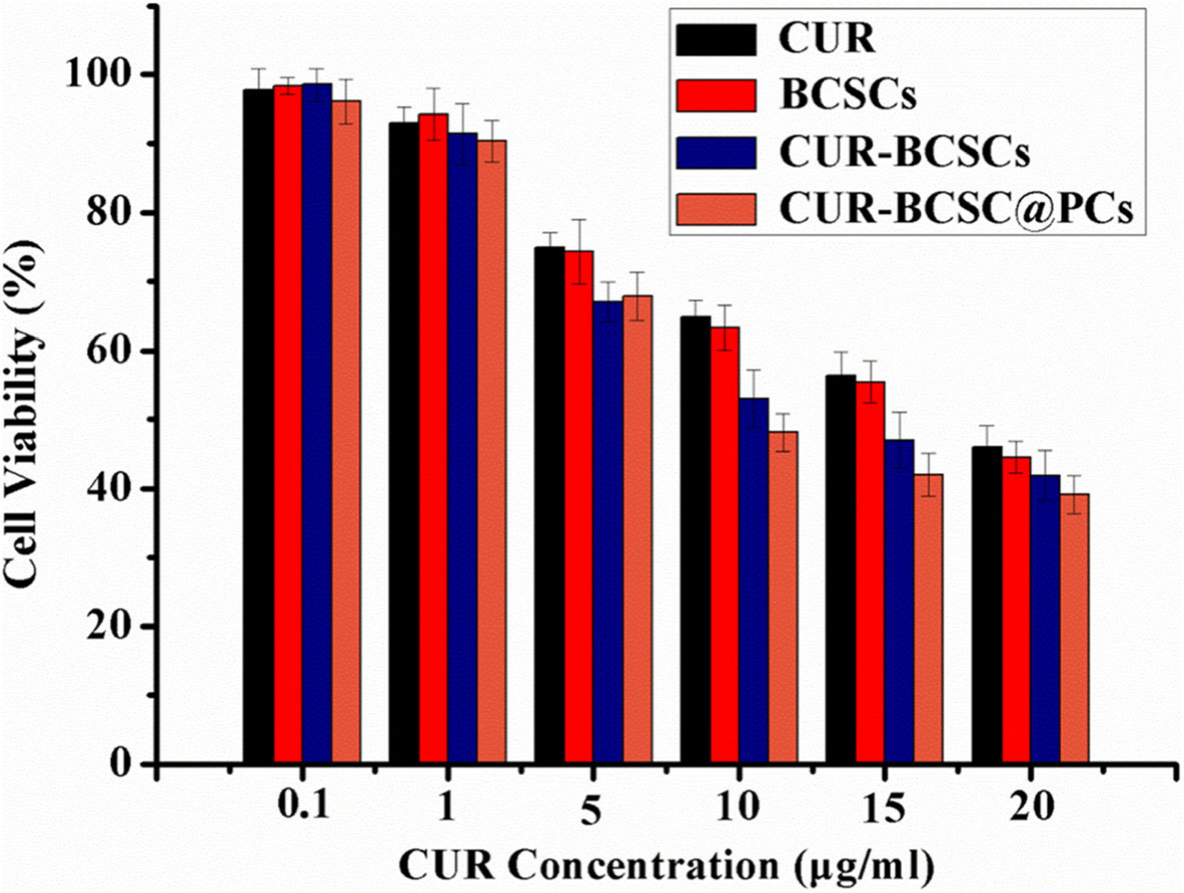
In vitro cytotoxicity of different formulations at 24 h in A549 cells

MTT assay was conducted to investigate the cytotoxic effects of free CUR, BCSCs, CUR-BCSCs, and CUR-BCSC@PCs on A549 cell lines [35]. The cell viability data is summarized in Fig. 8. All the CUR preparations showed dose dependence in terms of cell proliferation inhibition. As shown in Fig. 8, free CUR had slightly less capability for inhibiting cellular proliferation against all A549 cells compared with CUR-BCSC@PCs and CUR-BCSCs, after incubation for 24 h. For A549 cells, the anti-cancer activity of CUR-BCSC@PCs was better than that of BCSCs and CUR-BCSCs, which was likely due to excellent cell uptake. Furthermore, CUR-BCSC@PCs showed a higher cytotoxicity property than CUR-BCSCs, indicating that PC has a potential inhibitory effect on the proliferation of A549 cells.

### In vitro Cell Uptake Study

**Fig. 9:**
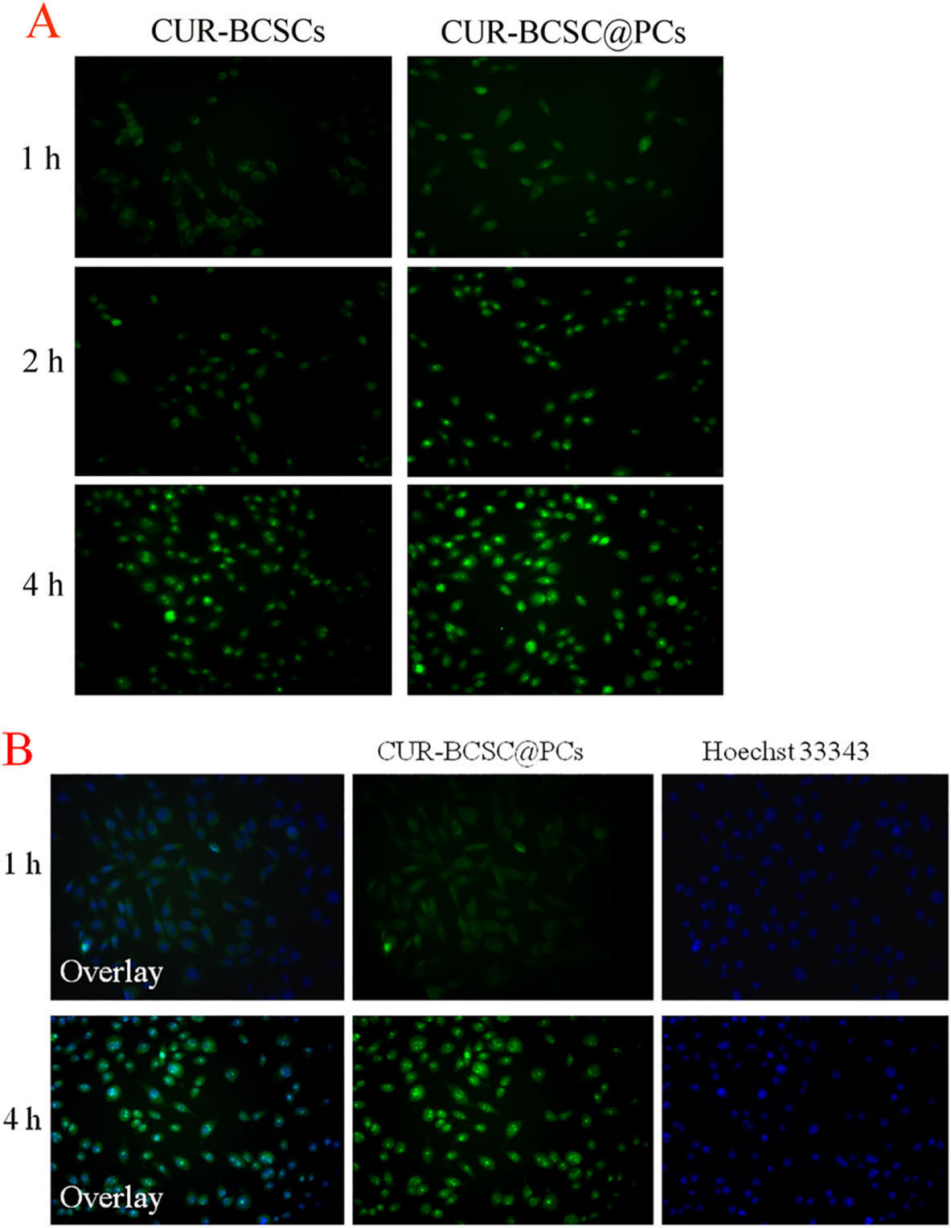
**a** Fluorescent imaging of the cellular uptake of CUR-BCSCs and CUR-BCSC@PCs at different times. **b** The cell location of CUR-BCSC@PCs at 1 h and 4 h

As shown in Fig. 8, the fluorescence signals of CUR were observed in A549 cells treated with CUR-BCSCs and CUR-BCSC@PCs (CUR: 20 μg/mL) at 1, 2, and 4 h, using the inverted fluorescence microscope. CUR, as a green fluorescence probe, was also an anti-cancer drug, which was frequently used as a hydrophobic model drug to develop new and efficient drug delivery systems. As illustrated in Fig. 9a, both CUR-BCSCs and CUR-BCSC@PCs were absorbed in A549 cell lines, and the uptake efficiency was time-dependent. Owing to the over-expressed biotin receptors on the surface of cancer cells, biotin-loaded nano-micelles had an affinity for A549 cells. The fluorescence signals from CUR-BCSC@PCs were high at 4 h, indicating a high cell uptake rate of CUR-BCSC@PCs. The fluorescence signals of CUR-BCSC@PCs were higher at 4 h than that at 1 h or 2 h; this proved that the cell uptake was time-dependent.

The nuclei of A549 cells were stained by Hoechst 33342. As shown in Fig. 9b, green fluorescence signals were seen in the cytoplasm of the 1 h group of CUR-BCSC@PCs, and the fluorescence gradually occurred in the nuclei of the 4 h group of CUR-BCSC@PCs, which demonstrated cellular uptake through caveolae-mediated endocytosis.

## Conclusions

In this study, a type of protein-functionalized COS nanoparticle was prepared through condensation reactions, self-assembling behavior, and the interaction between PC and CUR-BCSCs. After the preliminary investigation, the amphiphilic carrier materials (BCSCs) with redox sensitivity were synthesized successfully and verified using ^1^H-NMR. The surfaces of CUR-BCSCs were modified with a layer of phycocyanin corona, which could improve the cell uptake efficiency and protect CUR-BCSC@PCs from plasma protein adsorption. The cellular cytotoxicity and uptake analysis indicated that CUR-BCSC@PCs could transport CUR into the A549 cells and has an excellent anti-proliferative property. Considering the PDT effect of PC and CUR, we will evaluate photodynamic properties and anti-cancer activity of CUR-BCSC@PCs under phototherapy in the next phase of this research. This study paved the way for the improvement of anti-cancer drug efficacy and the introduction of a functional protein corona. In summary, the nanomedicine carrier biomaterial of CUR-BCSC@PCs based on COS with multiple functions has provided a new strategy for tumor treatment and exhibited great application prospects.

## Data Availability

The conclusions made in this manuscript are based on the data which are all presented and shown in this paper.

## Abbreviations

BCSC: Biotin-chitosan oligosaccharide-dithiodipropionic acid-curcumin
COS: Chitosan oligosaccharide
COS-S-S-CUR: Chitosan oligosaccharide-dithiodipropionic acid-curcumin
CUR: Curcumin
CUR-BCSC@PCs: Phycocyanin-functionalized and curcumin-loaded biotin-chitosan oligosaccharide-dithiodipropionic acid-curcumin micelles
CUR-BCSCs: Curcumin-loaded biotin-chitosan oligosaccharide-dithiodipropionic acid-curcumin micelles
EPR: Enhanced permeability and retention
PC: Phycocyanin
PDT: Photodynamic therapy
